# Mobile Apps to Support Family Caregivers of People With Alzheimer Disease and Related Dementias in Managing Disruptive Behaviors: Qualitative Study With Users Embedded in a Scoping Review

**DOI:** 10.2196/21808

**Published:** 2021-04-16

**Authors:** Marjorie Désormeaux-Moreau, Charlie-Maude Michel, Mélanie Vallières, Maryse Racine, Myriame Poulin-Paquet, Delphine Lacasse, Pascale Gionet, Melissa Genereux, Wael Lachiheb, Véronique Provencher

**Affiliations:** 1 School of Rehabilitation Faculty of Medicine and Health Sciences Université de Sherbrooke Sherbrooke, QC Canada; 2 Institut universitaire de première ligne en santé et services sociaux Centre intégré universitaire de santé et de services sociaux de l'Estrie Centre hospitalier universitaire de Sherbrooke Sherbrooke, QC Canada; 3 Department of Community Health Sciences Faculty of Medicine and Health Sciences Université de Sherbrooke Sherbrooke, QC Canada; 4 Public Health Directory Centre intégré universitaire de santé et de services sociaux de l'Estrie Centre hospitalier universitaire de Sherbrooke Sherbrooke, QC Canada; 5 Research Center on Aging Centre intégré universitaire de santé et de services sociaux de l'Estrie Centre hospitalier universitaire de Sherbrooke Sherbrooke, QC Canada

**Keywords:** disruptive behaviors management, dementia, caregivers, mobile phone, app, scoping review, focus group, mHealth, neurocognitive disorder

## Abstract

**Background:**

People with Alzheimer disease and related dementias often display disruptive behaviors (eg, aggression, wandering, and restlessness), which increase family caregivers’ burden of care. However, there are few tools currently available to help these caregivers manage disruptive behaviors. Mobile apps could meet this need, but to date little is known about them.

**Objective:**

The aims of our study were to identify existing mobile apps designed to support family caregivers of people with Alzheimer disease and related dementias in managing disruptive behaviors; explore whether family caregivers view these mobile apps as relevant to meeting their needs and as useful in managing disruptive behaviors; and document the types of mobile apps that are of interest and appeal to most family caregivers (with regard to format, ergonomics, and clarity).

**Methods:**

A review of mobile apps initially conducted in February 2018 was updated in March 2019 with 2 platforms (App Store [Apple Inc.] and Google Play [Google]). The selected apps were first analyzed independently by 3 raters (2 students and 1 researcher) for each of the platforms. A focus group discussion was then held with 4 family caregivers to explore their perceptions of the apps according to their needs and interests. The content of the discussion was analyzed.

**Results:**

Initially, 7 of 118 apps identified met the inclusion criteria. An eighth app, recommended by one of the knowledge users, was added later. Four family caregivers (women aged between 58 and 78 years) participated in the discussion. Participants expressed a preference for easy-to-understand apps that provide concrete intervention strategies. They reported being most inclined to use two apps, Dementia Advisor and DTA Behaviours.

**Conclusions:**

Few mobile apps on the market meet the needs of family caregivers in terms of content and usability. Our results could help to address this gap by identifying what family caregivers deem relevant in a mobile app to help them manage disruptive behaviors.

## Introduction

### Background

Due to the aging population, an increased prevalence of dementia is expected in many countries over the coming years [1]. In Canada, it is estimated that dementia will affect around 674,000 people by 2031, which is almost twice as much as the number in 2011 [2]. People with Alzheimer disease and related dementias (ADRD) often display disruptive behaviors, such as aggression (behavioral or verbal), wandering, and agitation (excessive or inappropriate verbal or motor behaviors) [3,4]. About 50%-70% of the people with ADRD live at home and require increasing care as the disease progresses [5,6]. Managing disruptive behaviors can thus present real challenges for family caregivers. Although taking care of people with ADRD may often have a positive effect on caregivers (eg, sense of personal accomplishment and growth) [7], they remain at greater risk of suffering from negative psychological (eg, anxiety, depression), emotional, and physical (eg, intense fatigue, other health problems) consequences, as well as from financial issues and job loss [8]. Informal caregiving represents up to almost half of the care provided to people with dementia [9]; therefore, helping family caregivers to lower the frequency of disruptive behaviors, promoting their self-efficacy to manage these behaviors, and minimizing their stress when they occur is crucial.

Many technological tools are available to improve the quality of life of people with ADRD and to reduce the mental and emotional burden felt by family caregivers by helping them with the care, treatment, and management of the disease [10]. For example, GPS technologies, including tracking devices (eg, wandering path tracking and fall detection) [11] and intelligent interface devices (eg, Stay in Touch) [12] can help to locate the person with ADRD and to communicate with the family caregiver in case of emergency. Additionally, platforms offering informal support to family caregivers through sensors located in the home that can monitor the behaviors of the person with ADRD (eg, iCarer [13], passive remote patient monitoring [14], QuietCare [15]) may represent possible solutions for family caregivers to improve the care provided [13]. Finally, online communities have been created for family caregivers, which may reduce isolation [16] and support the sharing of experiential knowledge and skills [16,17].

These technologies are often reported to be complicated to use by caregivers or to require intensive and sustained training [18]; nonetheless, the increasing use of smartphones has generated considerable growth in the development of mobile apps in the health sector, including for people with ADRD. These apps mainly aim at improving the cognitive functions of the person with ADRD while maintaining social interactions [19]. Some of these apps can help reduce the anxiety of family caregivers by monitoring the person in and around the home in real time, estimating the probability of wandering using geolocation, as well as facilitating care management and services by health care professionals [20]. Based on data collected by the mobile device, some apps also offer security options, such as calling emergency services, guiding the person to a safe place (using Google navigation) or informing family caregivers of the geographic location of the person with ADRD.

Although several mobile apps have been designed for people with ADRD, very few are specifically designed to be used by their family caregivers [19] with these primarily being conceived to monitor the location or activities of daily living [21] of the person with ADRD. However, family caregivers have also expressed other important needs, namely the management of their loved one’s mood and disruptive behaviors [22]. It would thus be relevant to explore whether there are simple, credible, and accessible mobile apps that meet these needs [23]. Mobile apps have the potential to reach many family caregivers, as the majority use smartphones more than computers [23]. Being easy and quick to update, they allow family caregivers to access the most recent data [19]. Mobile apps are reported to be a more effective tool than conventional methods, such as classroom training, to inform caregivers about ADRD [23]. They could therefore be relevant and handy tools to promote learning and knowledge among family caregivers of people with ADRD. Supporting them in managing disruptive behaviors is essential if they are to increase their sense of competence or self-efficacy, which may in turn reduce their burden of care and improve their psychological well-being.

### Context of the Study and Objectives

In fall 2017, the researchers (VP and MDM) were approached by a nonprofit organization (the Quebec chapter of the international Planetree network) with expertise in implementing best practices based on a person-centered approach. This organization wanted to adapt a Dutch mobile app to the Quebec context in order to reduce caregivers’ burden by helping them manage disruptive behaviors of people with ADRD. However, a review of similar available mobile apps was deemed to be considered necessary prior to adapting the Dutch mobile app. The aim of this study was to provide family caregivers with a mobile app that could help them manage disruptive behaviors and thus reduce their burden of care. The following specific objectives were jointly defined by the local director of public health (MG), the director general of the Quebec chapter of the international Planetree network, and VP and MDM: identify existing mobile apps designed to support family caregivers of people with ADRD in managing disruptive behaviors; explore the family caregivers’ view of these mobile apps regarding their relevance to meet their needs and their usefulness in managing disruptive behaviors; and document the types (eg, format, ergonomics, clarity) of mobile apps that are of interest and appeal to the most family caregivers.

## Methods

### Design

An increasing number of studies have been published in recent years aimed at identifying and analyzing mobile apps available on the market in various health disciplines [23-26]. To use a structured and systematic framework consistent with our objectives, a scoping review [27] was conducted. Although scoping reviews traditionally involve research studies, the method seemed appropriate for identifying apps available on the market and for targeting those which may support family caregivers of people with ADRD in managing disruptive behaviors. Scoping reviews may indeed provide an overview of the available documentation to examine the extent of the current knowledge on a particular subject [27]. The selected approach was based on the following 6 steps described by Arksey and O’Malley [27] and revised by Levac et al [28]: (1) formulation of research questions, (2) identification of relevant sources, (3) selection of relevant mobile apps, (4) data extraction and organization, (5) data analysis and results synthesis, and (6) consultation.

### Formulation of Research Questions

This scoping review aimed to answer the following research questions: (objective 1) What mobile apps are available to support family caregivers of people with ADRD in managing disruptive behaviors and what are their characteristics? (objectives 2 and 3) Do these mobile apps meet the needs of family caregivers (ie, perceived relevance and usefulness) and arouse their interest in using them?

### Identification of Relevant Sources

The search strategy was established by 5 occupational therapy students (PG, DL, CMM, MPP, MR, and MV) and validated by 2 researchers (VP and MDM). The search was conducted from February 21 to February 28, 2018, on the most popular commercial app stores, Google Play Canada (Google) and App Store Canada (Apple Inc), using the following keywords: “Démence,” “démence proche aidant,” and “Alzheimer proche aidant” in French; and “Dementia,” “dementia caregiver,” “Alzheimer,” and “Alzheimer caregiver” in English. Two models of smartphones were used, a Samsung Galaxy A5 and an iPhone SE (Apple Inc), with Android (Google) and iOS (Apple Inc) operating systems respectively.

### Selection of Relevant Mobile Apps

App inclusion criteria for apps included the following: French or English language; the targeting of disruptive behaviors associated with ADRD; a main function of informing, educating, or equipping family caregivers of people with ADRD; and free use. Meanwhile, the exclusion criteria for apps were those with an exclusive focus on psychological support for family caregivers or the screening for early signs and symptoms of ADRD, and those that required payment.

Two occupational therapy students (DL and CMM) first identified the mobile apps based on the titles. A minimum of 50 applications per store was first selected to ensure a good diversity in the results. After reaching this threshold, searches were continued until 10 consecutive applications no longer met the criteria (eg, memory game app for entertainment and not in conjunction with some cognitive stimulation to prevent the onset of ADRD) in order to ensure that as many relevant apps as possible were identified. DL, CMM, VP, and MDM then screened the relevance of the first identified apps to determine if they met all the inclusion criteria and did not meet any of the exclusion criteria based on their description. Apps common to both stores were identified and counted only once. When the search was updated (March 10-15, 2019), the apps that no longer satisfied the eligibility criteria were removed.

### Data Extraction and Organization

The mobile apps selected in the previous step were then downloaded and organized in a Microsoft Excel data chart developed by the research team according to the following information: app name and download size description of the interface, internet connection required to access content (once the app has been downloaded); and content of the app (categories of information and how information is presented). The data were organized following two parallel processes, one for mobile apps identified in Google Play (coordinated by DL) and the other for App Store (coordinated by CMM). Apps that no longer satisfied the inclusion criteria following this in-depth analysis were excluded. In case of uncertainty, VP was consulted to validate the decision. The suggested Dutch app (Dementiegame) by Planetree network was subsequently included in the process.

### Data Analysis and Results Synthesis

Data analysis was based on a qualitative and iterative process. The information collected was extracted to a grid based on the following predetermined themes: quality (credibility and accuracy of the information), accessibility and comprehensibility, and usability (speed and complexity). They were inspired by the themes central to the concepts of translational validity, which includes both face validity and content validity [29] and evolved throughout the process.

All the selected apps were analyzed independently by 3 raters (2 students and a researcher), for each of the app stores, Google Play (DL, MV, and MDM), and App Store (CMM, PG, and MDM). The apps were assessed by each rater according to their relevance, and disparities were resolved by consensus.

### Consultation With Knowledge Users

A focus group meeting was conducted with family caregivers of people with ADRD (knowledge users) to explore their perceptions of the selected apps according to their needs and interest in using them. This method is more suitable for exploring the positive and negative components in usability and usefulness of new technology, crossing perspectives, and gaining more detailed feedback (generated by sharing information between the different participants) than are one-on-one single interviews [30].

### Recruitment and Selection of Participants

Participants were recruited using a purposive nonprobability sampling technique (29). We presented the study (objectives and main stages of achievement) to caregivers (N=30) who attended meetings held by 2 community support organizations. A brief description of the study and the contact details of the person to reach were given to family caregivers interested in participating in the research project. To be included in the study, participants had to be a family caregiver (eg, husband, wife, daughter, son) of a person with disruptive behaviors associated with ADRD, speak French and have a good understanding of written French and English, and have concerns about disruptive behaviors exhibited by a family member with ADRD.

### Data Collection (Focus Group)

Data were collected during a face-to-face focus group meeting at the Research Center of Aging. The meeting began with a presentation of the selected apps to the participants, who had received them a week prior to the focus group in order to allow for some familiarization. This presentation was made by MPP and PG to help caregivers understand the aim of the apps. The focus group, led by a researcher trained in the qualitative approach (MDM), allowed the participants to comment on the perceived relevance of the apps’ content (useful information, meets users’ needs) and their interest in future use. Participants were also encouraged to comment on issues or questions that had not been addressed. Evidence from previous studies [31,32] inspired the development of the focus group guide (Multimedia Appendix 1). The meeting lasted 94 minutes. The discussion was digitally audio-recorded and then fully transcribed by CMM and MR. Participants were also asked to complete a sociodemographic questionnaire documenting their age and gender, their relationship with and level of involvement in the care of the person with ADRD, and the type of mobile phone they used.

### Data Analysis (Focus Group)

Transcription of the focus group meeting was content analyzed [33]. MV, DL, and MDM manually and independently coded the data using a grid with the predetermined themes of relevance to participants’ needs and perceived usefulness. Participants’ comments were first associated with these themes and then inductively subdivided into categories and subcategories as the analysis progressed. The coding and categorization were carried out independently and then corroborated by all members; if a discrepancy arose, the issue was discussed to reach a consensus.

The local director of public health (MG) and the director general of the Quebec chapter of the Planetree network were consulted during the process to ensure the relevance of the results and the selection of the most efficient knowledge transfer strategies. Meetings with members of the research team were held on a quarterly basis (2018) and then annually (2019, 2020).

### Ethical Considerations

The study was approved by the ethics committee of Centre intégré universitaire de santé et de services sociaux de l’Estrie, Centre Hospitalier Universitaire de Sherbrooke. Participants completed a consent form before participating in the focus group.

## Results

### Selection of the Relevant Mobile Apps

**Figure 1:**
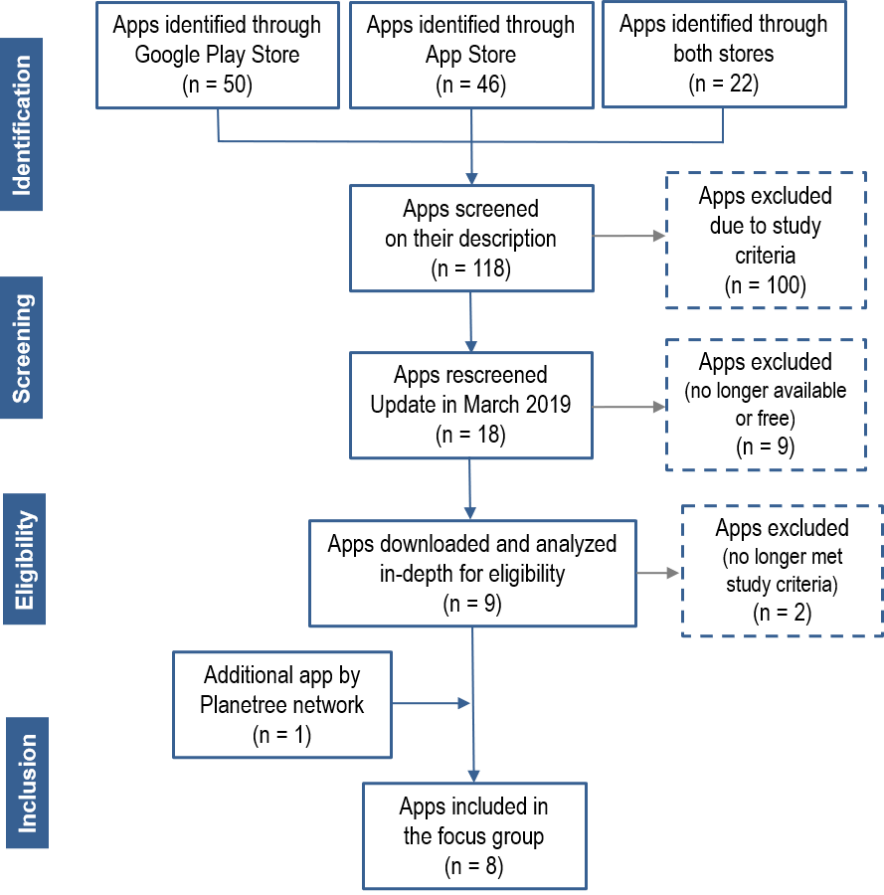
Flowchart of the app selection process.

Figure 1 shows the flowchart of the app selection process. The searches in Google Play and App Store identified 118 apps (22 available on both platforms, 50 available only in Google Play, and 46 available only in App Store) based on title screening. Their descriptions were then screened based on the inclusion and exclusion criteria. Apps were mainly excluded due to their aim not being in line with the research objectives, and several involved only disease screening, games, therapy, or even fundraising. Others were designed to help the person with ADRD to function and were based on functionalities that were not relevant to the present study (geolocation, management of schedules, alarm, etc). Finally, several apps did not target disruptive behaviors or did not provide tools to support the care provided by family caregivers (detailed descriptions of dementia types, causes, and symptoms). The population targeted by the apps was another reason for exclusion. An app could target several populations. Ultimately, 18 of the initially identified 118 apps (15.3%) remained after applying the inclusion and exclusion criteria.

Following the update in March 2019, half of these 18 apps (n=9) were excluded because they were no longer free or no longer available. In addition, 1 app no longer targeted disruptive behaviors, and another only referred users to a website. In the end, 7 apps were eligible for the focus group. By adding the app suggested by the director general of the Quebec chapter of the Planetree network, 8 apps were ultimately included in the analysis: 1 was only available on Google Play, 1 on App Store, and 6 were available on both platforms.

### Description of the Relevant Mobile Apps

Of the 8 apps selected, 7 were in English, and only 2 of these, Dementia Advisor (English and French) and Dementia Support (English, German, Dutch, and Portuguese) were available in more than one language. The Dutch app, Dementiegame, was only available in Dutch and was the only app in the form of an interactive game. Multimedia Appendix 2 provides a description of the 8 apps that were presented to the family caregivers during the focus group meeting.

### Consultation With Knowledge Users: Description of Participants

Four family caregivers of people with ADRD showed interest in the study and took part in the focus group meeting. Table 1 presents the characteristics of the sample. Participants were all French-speaking White women aged from 58 to 78 years. Two daughters and two spouses acted as the main family caregivers with an active and daily involvement with the person with ADRD. Participants were at different levels of caregiving, with relatives at the beginning, the middle, and the advanced stage of the disease. Only 1 participant had a deceased relative; however, she had an extensive caregiving experience with her husband and remained active in her caregiver role by supporting other loved ones. The participants were evenly distributed between the 2 types of devices (Android or Apple) and their familiarity with the device was varied (quite to very familiar).

**Table 1:** Characteristics of focus group participants (n=4).

Participant	Age (years)	Relationship with the person with ADRD^a^	Intensity/frequency of interactions with the person with ADRD	ADRD stage	Mobile device/familiarity with it
1	58	Daughter	Active, daily	Advanced	Android/familiar
2	67	Daughter	Active, daily	Deceased	iPhone/very familiar
3	70	Spouse	Active, daily	Beginning	Android/quite familiar
4	78	Spouse	Active, daily	Middle	iPad/quite familiar

^a^ADRD: Alzheimer disease and related dementias.

### Consultation With Knowledge Users: Relevance and Perceived Usefulness of the Mobile Apps

Participants were asked if the selected mobile apps met their needs and aroused their interest. Their feedback was divided into 2 themes: (1) relevance of the mobile apps and (2) perceived usefulness of the mobile apps. A total of 13 categories and 13 subcategories were identified in relation to these 2 themes. Multimedia Appendix 3 (relevance) and Multimedia Appendix 4 (perceived usefulness) summarize the results.

#### Relevance of Mobile Apps

Participants spontaneously rated the relevance of the mobile apps regarding the fit (or not) between content and perceived needs. Participants identified 3 mobile apps (DTA Behaviours, Dementia Advisor, and Dementia Emergency) that they thought contained the information that family caregivers may need to manage disruptive behaviors at a given point in the course of the disease. Referring to the DTA Behaviours app, one participant said, “Well, all the subjects that are named, I mean in any case, I went through ALL of them with my mother, ALL […] at different stages”. Participants also noted that certain apps (Dementia Games and Dementia Emergency) did not seem relevant to supporting a caregiver in managing disruptive behaviors, as their content was more about actual changes in behaviors with ADRD than advice on how to deal with them. They also thought that some apps might be more helpful to other family members less involved in care than the main caregiver; for example, one participant said, “But on the other hand, the last one you presented to us [Dementiegame], I see [it] [as being for] children or brothers and sisters.” Indeed, these apps relate more to the impact of the disease on daily living, a reality that other family members are less aware of as compared to the main caregiver.

#### Perceived Usefulness of Mobile Apps

First, participants were asked about the likelihood of their using the mobile app and about the context in which they might be more likely to use them. Not surprisingly, compatibility of the apps with their mobile device was the main factor influencing use. Furthermore, participants expressed little interest in certain apps (Care4Dementia and Alzheimer’s Daily Companion) because they did not see any added value:

It’s […] like a book. No need for a mobile application [to present such content], it’s like […] I press here and it brings me [to a text], and [if I press there, it brings me back to another text]. Why not get a book and leave it at that?

Participants recommended using certain apps (DTA Behaviours, Dementia Support, and Dementia Advisor) before disruptive behaviors occurred. One participant expressed this idea in reference to the Dementia Advisor app: “The first thing I would do is look at all the daily situations presented, with a cool head.” In addition, the apps may be used afterwards (for example, Dementia Advisor and DTA Behaviours) to get feedback on their interventions. As one participant said, “After the situation gets better also, but here I would go to see what I did, what I could have done better.” Other apps seemed interesting in terms of using them on the spot when needed: “If my mom has a terrible attack, [I can open] my app […], then I’ll see what I can do […], ok well. […] It’s like here and now.” Also, not surprisingly, participants said they were more attracted to easier-to-understand apps, especially those available in their mother tongue (ie, in French vs only in English). Family caregivers reported being inclined to use mobile apps when the information was clear, even if not in French (ie, DTA Behaviours, Dementia Advisor, and Dementia Emergency). The format of the mobile apps and, more precisely, the way in which the information was presented and organized, also influenced their opinion:

But […] the first one I had earlier, on [Android, Dementia Emergency], well I understood it very quickly […] I was not lost at all

because the information is well organized and easy to find

Conversely, participants were less likely to use mobile apps that required more steps to find needed information; for instance, one participant did not like Care4Dementia because there was “too much research.”

### Overall Rating

**Figure 2:**
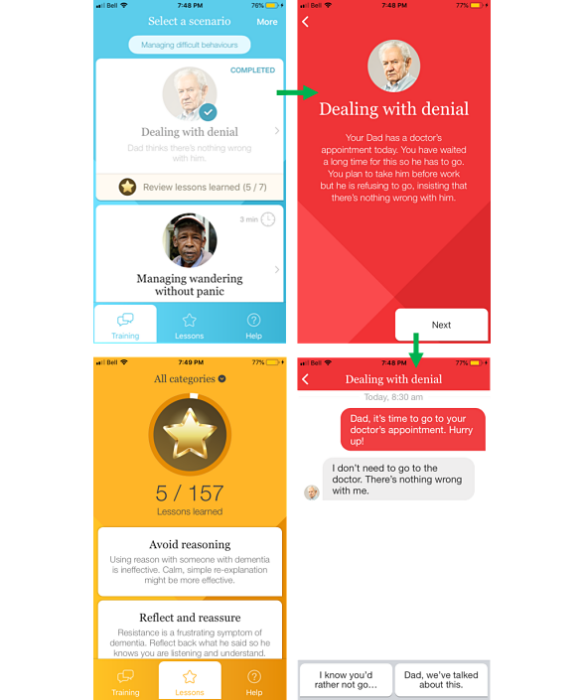
Screenshots from the Dementia Advisor app.

**Figure 3:**
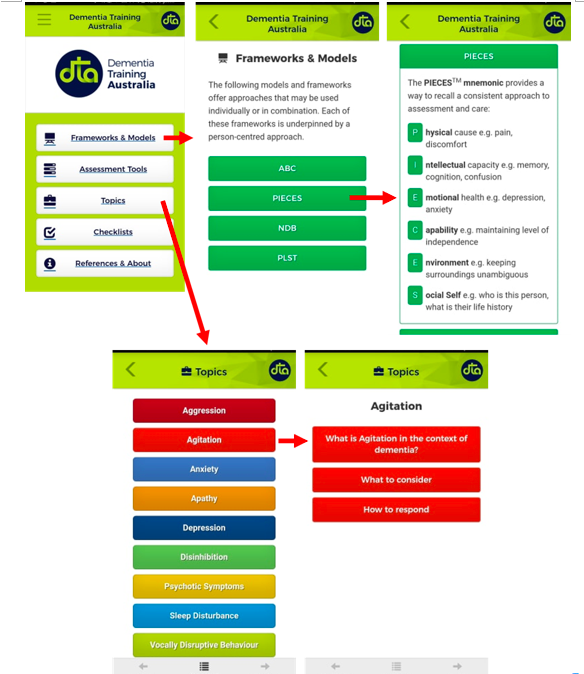
Screenshots from the DTA Behaviours app.

Participants concluded that the ideal mobile app would include concrete intervention strategies to apply when disruptive behaviors occur. In this regard and based on the apps themselves (regardless of the platform/mobile device), participants reported that they could potentially use 2 of the apps, Dementia Advisor and DTA Behaviours. As one said, “These are tools that I need, really concrete: there is a behavior [which arises and the application tells you] and what you can do [to cope with it]”. Figure 2 and Figure 3 show sample screenshots of Dementia Advisor and DTA Behaviours, respectively.

## Discussion

### Main Findings

This study aimed to identify currently available mobile apps in Canada developed to support family caregivers in managing disruptive behaviors of people with ADRD, explore the relevance and usefulness of these apps as perceived by caregivers, and document the types of apps that appeal to the most family caregivers. Of the 118 apps inventoried, 8 were selected to be reviewed by caregivers, and only 2 of these were perceived as relevant and useful by caregivers.

Our review suggested that there are currently a limited number of mobile apps on the market targeting family caregivers to help them deal with disruptive behaviors of people with ADRD. Because health apps only started expanding in 2013 [34], customized apps to assist caregivers in dealing with disruptive behaviors of people with ADRD are still scarce. Moreover, for the apps reviewed, it was challenging to determine if the information provided was evidence-based and to what extent it met family caregivers’ needs.

According to our results, few mobile apps sufficiently met the caregivers’ needs in managing disruptive behaviors. More specifically, only Dementia Advisor and DTA Behaviors appealed to most of the participants by offering concrete strategies to manage disruptive behaviors of people with ADRD. Participants mentioned that these apps also have a well-organized design interface, providing customized and clear information for quick searches. As most users, including caregivers, now use smartphones [34], it is important to prevent apps from being difficult to use [35]. Past studies reported that the small size of smartphone screens and texts in the apps were common usability issues, especially for older caregivers [36,37]. Hence, app usability is a key factor that needs to be addressed to improve caregivers’ experience [38]. Overall, our findings are in line with previous results as they highlighted the importance of adapting health apps to the needs of users, including caregivers [34].

Regarding the Dementiegame app, most participants said that it did not meet their current needs, due to difficulty navigating through the app and accessing information. Our results underline the importance for future studies to involve family caregivers in designing useful, relevant, and easy-to-use apps, especially by providing concrete strategies to help them deal with disruptive behaviors on a daily basis. In this regard, the ‘living lab” approach might be adopted since it aims to develop innovative, sustainable solutions to the growing challenge of managing disruptive behaviors of people with ADRD [39]. 

Finally, our review of mobile apps was updated in May 2020, using the same 2 platforms (App Store and Google Play). One new relevant app called CogniCare was found. This app was updated recently in April 2020. It provides a rich source of useful tips and short videos to help family caregivers manage disruptive behaviors of their loved ones with ADRD. As our study and content analysis of the focus group discussion was completed before we found this new app, our results only apply to the apps previously reviewed in this paper.

### Strengths and Limitations

This study has several strengths. First, for the scoping review, we followed a rigorous, reliable approach based on Levac [28]. Many scoping reviews do not include the last step (consultation), but we performed it using a rigorous method to validate the results with family caregivers. The diverse profiles of the family caregivers who participated in the focus group discussion was a strength of this study (children and spouses of various ages). Second, the analysis of the selected mobile apps, exploration of the app once downloaded, and data validation by 4 team members (2 per type of mobile app store) contributed to the study’s reliability and reduced subjectivity bias. Finally, the study included only free apps. Although this decision may limit the number of apps, this methodological choice was deemed essential by the local director of public health to increase access to the general public, especially to caregivers with financial issues.

The study also has some limitations. First, as apps were searched for on the App Store Canada and Google Play Canada databases, the results only reflect the app market in this country. It was also not possible to cover all existing apps. Thus, an arbitrary limit was placed on our search. As a result, some apps could have been omitted, even though the cutoff used suggests that few relevant apps would have met the inclusion criteria. Third, some apps used external websites. As the information provided outside of the apps was not reviewed as thoroughly as was the in-app content, we do not know the quality of the content provided to family caregivers through these external links. Further studies should ensure that the applications developed to support caregivers of people with ADRD are based on evidence-based data (eg, theories of managing behavioral symptoms). Furthermore, the number of family caregivers in our focus groups was small, and no male caregivers could participate in the study within the timeframe of recruitment. Despite the group being all women, the 4 participants were varied in terms of age (58 to 78 years), relationship with the person with ADRD (2 spouses and 2 daughters), and literacy level. It is not surprising to have recruited only women, as around two-thirds of caregivers of people with dementia are women [40]. Moreover, the majority of persons who attended the meetings held by key community support organizations (where we recruited) were women. It is also well known that elderly women are more likely to participate in research studies than their male counterparts. Several studies carried out with caregivers of patients with dementia have mainly women as participants [41,42]. Although few in number, the participants had different levels of familiarity with the technology (from quite to very familiar) and had a rich experience of caregiving. Finally, the themes emerging during the focus group discussion triggered an emotional reaction in some participants, who were not comfortable discussing the app. Therefore, providing time at the outset to address emotional issues might have allowed participants to vent their emotions and then focus on the study’s objectives. Recruiting former caregivers may provide access to rich experience while reducing the likelihood of being emotionally overloaded during the study.

### Recommendations and Future Directions

The focus group discussion helped to identify what family caregivers find relevant and useful in a mobile app, even if future studies should involve more participants. Inclusion of concrete intervention strategies appears to be an important feature. These findings may guide the development of future apps for these caregivers. In addition, using mobile apps is an effective way to improve knowledge because they are ready at hand and can be consulted quickly. Apps are therefore likely to reduce difficulties, such as being afraid of leaving the family member alone at home, encountered by many current training courses. On the other hand, technological difficulties can impede their use, which underlines the importance of involving family caregivers with different degrees of digital literacy when designing apps [43]. In addition, as the medical terminology used in apps should be easy for target users to understand [44], future studies should determine to what extent the apps are comprehensible to caregivers with differing degrees of health literacy, a factor which was not fully examined in our study.

One important public health priority is to promote access to knowledge tools for every individual, especially the most vulnerable. In this regard, some of the apps reviewed require an internet connection, which may reduce caregivers’ access to them, as not everyone can afford internet services. Moreover, most of the apps reviewed did not have password protection or require login. One common concern of mobile health apps is privacy, as users often enter their loved ones’ health information [45,46]. Future attention should be paid to ways to protect users’ private information, without this being a barrier to using the app.

Finally, with the constantly evolving market, a certain “volatility” of the available apps has been noted. In fact, some of the apps identified might have been discontinued, while new ones may have appeared. There are also variations between the 2 stores regarding available apps. It is therefore suggested that an app search be carefully planned to ensure exhaustivity and reproducibility with respect to the review of the apps. Indeed, 2 people may not find the same list of apps in Google Play due to the algorithms used to partially personalize the results [47]. In addition, we must remain critical about the list generated, as sponsored apps top the list despite not necessarily being the most relevant.

### Conclusions

Considering the proliferation of mobile apps and their increased use by family caregivers, available mobile apps designed to help manage disruptive behaviors should meet their needs in terms of both content and usability. However, when this study was conducted, few apps met these criteria. Therefore, this study aims to reduce this deficiency by highlighting what caregivers consider relevant and useful in existing mobile apps, while identifying those tailored to family caregivers’ needs. These findings may help caregivers to manage disruptive behaviors more effectively and satisfactorily, reduce their burden of care and, ultimately, delay the institutionalization of people with ADRD.

## Appendix

Multimedia Appendix 1 Guide for focus group discussion.

Multimedia Appendix 2 Description of the eight selected apps presented to participants.

Multimedia Appendix 3 Emerging results for the relevance theme.

Multimedia Appendix 4 Emerging results for the perceived usefulness theme.

## Abbreviations

ADRD: Alzheimer disease and related dementias
