# Supplementary material for: Mobile Apps to Support Family Caregivers of People With Alzheimer Disease and Related Dementias in Managing Disruptive Behaviors: Qualitative Study With Users Embedded in a Scoping Review
Source: JMIR Aging. 2021 Apr 16;4(2):e21808. doi: 10.2196/21808 (PMC8087965; doi:10.2196/21808)
Supplement: Multimedia Appendix 1 [file aging_v4i2e21808_app1.docx]

**FOCUS GROUP - GUIDE**

**Perceived relevance of app content (useful information, meets users’ needs):**

1. Does the information provided by the app correspond to your reality as a family caregiver of a person with NCD?
   - - - What information would you like to find in the apps?
2. Do you feel that the app meets your needs in terms of understanding and managing disruptive behaviors?
3. Do you think that using one of these apps would make you feel better equipped to deal with your family member’s disruptive behaviors?
   1. In which cases do you plan to use one of these apps?
   2. To what extent would you recommend any of these apps to someone who could benefit from it?

**Appeal of and interest in future use (in particular, ergonomics, clarity and app’s format):**

1. What form of apps do you prefer: games, information, scenarios?
2. Which apps appealed to you the most? Why?
3. Which apps appealed to you the least? Why?
4. What is the easiest app to use? Why?
   1. What about the clarity of the information presented in the apps? Does one of the apps stand out in terms of clarity (comprehensibility) of the information?
5. Do you use any of these apps in your daily life? How often? Why?
