# Supplementary material for: Mobile Apps to Support Family Caregivers of People With Alzheimer Disease and Related Dementias in Managing Disruptive Behaviors: Qualitative Study With Users Embedded in a Scoping Review
Source: JMIR Aging. 2021 Apr 16;4(2):e21808. doi: 10.2196/21808 (PMC8087965; doi:10.2196/21808)
Supplement: Multimedia Appendix 2 [file aging_v4i2e21808_app2.pdf]

APPS PRESENTED TO PARTICIPANTS

App name<br>Version<br>(size-Mo)	Creation date<br>Last update	Interface description	Internet connection	App content	
				Categories of information	How information is presented
Dementia Advisor<br>V. 1.0.5.609<br>(26.12)	2017-06-02<br>2019-02-05	Home page shows 7 different categories and their themes.<br>The different themes are presented in the form of “text message” scenarios.	Not required to access content	According to the themes, 7 categories contain information for family caregivers:<br>- Manage difficult behaviors;<br>- Manage refusals;<br>- Manage family tensions;<br>- Not have time for oneself;<br>- Make difficult decisions;<br>- Manage work demands;<br>- Access and manage services.	Each category leads to a predetermined conversation with a person with NCD.<br>During the discussion, 2 response options are presented.<br>Lessons/explanations are provided during and at the end of the scenario.
Dementia Emergency<br>V. 1.2.<br>(30.4)	2016-01-12<br>2016-02-26	Home page shows 5 main categories (icons at the bottom of the page).<br>Home page also contains a tab with general tips (red icon in the center of the page).	Required to access content	According to the themes, 4 categories contain information for family caregivers:<br>- Emergency;<br>- Challenges;<br>- Assistance;<br>- General Information.	Each topic provides information in the form of lists and texts. It is best to read the “General Tips” section before starting to read the different categories because the app often refers to what was in this section.

App name<br>Version<br>(size-Mo)	Creation date<br>Last update	Interface description	Internet connection	App content	
				Categories of information	How information is presented
Alzheimer's Daily Companion<br>V. 1.0.7<br>(14.09)	2017-11-29<br>2018-04-20	Home page has 6 categories (icons in the center of the page) and 3 options (icons at the bottom of the page): notes, live assistance, and share.	Not required, except to click on Internet links	According to the themes, 6 categories contain information for family caregivers:<br>- Well-being;<br>- Communication;<br>- Behaviors / stay safe;<br>- Personal care;<br>- Activities;<br>- Research.	Each topic contains information in the form of lists, texts or Internet links.
Care4Dementia<br>V. 1.0<br>(58.9)	2015-03-15<br>No update	Home page has 4 categories and a search tab.	Not required to access content	4 categories contain information for family caregivers:<br>- General considerations and safety precautions;<br>- What happens to a person with dementia;<br>- Support for family caregivers;<br>- Background of family caregivers.	When a category is selected, it leads to a list of information with tips for family caregivers.<br>The "search" tab is used to search by keywords.
DTA Behaviours<br>V. 1.01<br>(22.51)	2017-08-31<br>2017-11-01	Home page has 5 categories (icons in the center of the page).<br>Each category contains themes and information.	Not required, except to click on Internet links	According to the themes, 5 categories contain information for family caregivers;<br>- Practice guides and models;<br>- Assessment tools;<br>- Topic;<br>- Control list;<br>- Reference.	The 2 most relevant tabs for family caregivers are Subjects and Reference guides/models.

App name<br>Version<br>(size-Mo)	Creation date<br>Last update	Interface description	Internet connection	App content	
				**Categories of information**	**How information is presented**
DementiAssist<br>V.1.3<br>(10.21)	2015-10-22<br>2016-08-24	Home page features 16 seniors’ photos representing consequences / behaviors of people with NCD.<br>Note: “category” has a typo and is written as “catagories”.	Not required to access content	16 consequences/behaviors of NCD are discussed: Aggression/anger, agitation/anxiety, apathy, confusion, walk hundred paces, problematic vocalization, psychosis, resistance to bathing, dressing, taking meals/ mouth hygiene, theft/search, inappropriate sexual behaviors, sleep problems and wandering.	Each category provides information in the form of texts for family caregivers.
Dementia Support<br>V. *n.a.*<br>(7.21)	2013-07-19<br>2017-04-25	Menu in the upper right used to navigate through the 9 categories	Not required to access content	Menu includes 9 categories: Welcome, inspiration, for family caregivers, support, dementia, network, about, settings and sharing.	For each theme, advice is given to help family caregivers to improve the quality of life, take care and facilitate daily activities of the person with NCD, and manage difficult situations.<br>This information is presented in the form of long texts.

App name<br>Version<br>(size-Mo)	Creation date<br>Last update	Interface description	Internet connection	App content	
				Categories of information	How information is presented
Dementiegame<br>V. 3.1<br>(908.6)	2016-03-21<br>2017-11-20	Comes in the form of an interactive game. Photos of elderly people are scattered across a city and, by clicking on the photos, various scenarios are presented. These deal with behaviors and consequences of NCD.	Not required, except to click on Internet links	Questions with choices of answers are presented during the scenario. Answering the questions correctly makes the person with NCD feels good.	Explanations are given in the form of a video at the end of each scenario.<br>3 scenarios include 4 levels, 2 include 3 levels, and 1 scenario includes 5 levels.
