# Supplementary figures and images for: Mobile Apps to Support Family Caregivers of People With Alzheimer Disease and Related Dementias in Managing Disruptive Behaviors: Qualitative Study With Users Embedded in a Scoping Review
Source: JMIR Aging. 2021 Apr 16;4(2):e21808. doi: 10.2196/21808 (PMC8087965; doi:10.2196/21808)

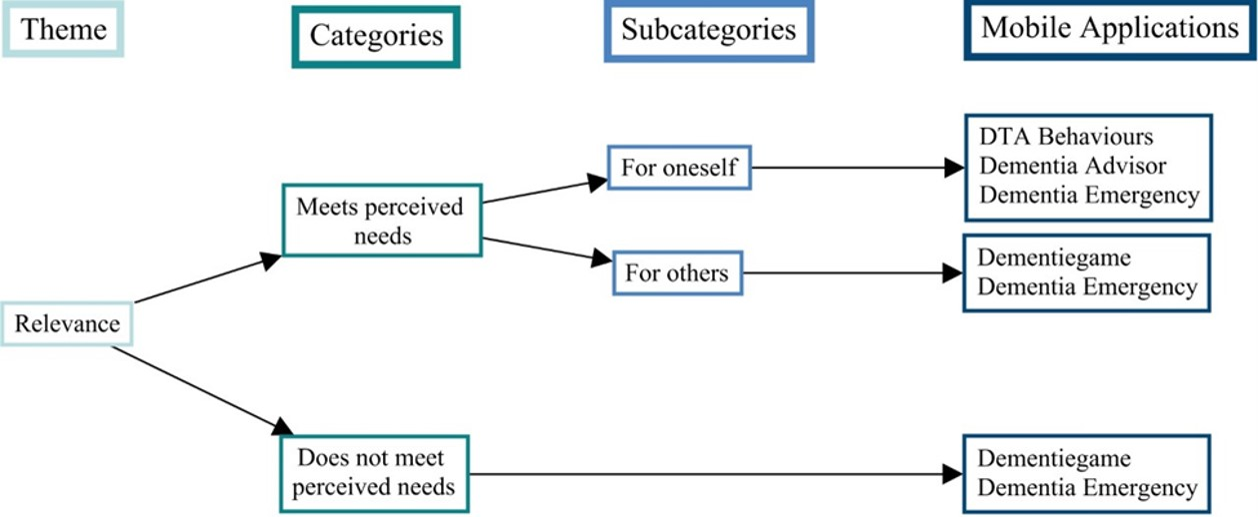

Supplement: Multimedia Appendix 3 [file aging_v4i2e21808_app3.png]

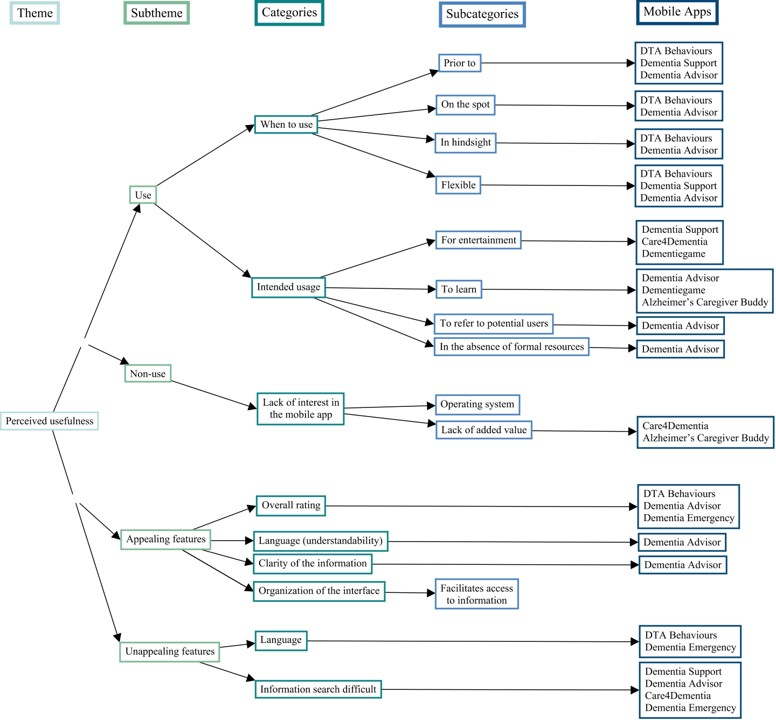

Supplement: Multimedia Appendix 4 [file aging_v4i2e21808_app4.png]
